# Impact of a mandatory geriatric medicine clerkship on the care of older acute medical patients: a retrospective cohort study

**DOI:** 10.1186/1472-6920-13-168

**Published:** 2013-12-16

**Authors:** Joye St Onge, George Ioannidis, Alexandra Papaioannou, Heather McLeod, Sharon Marr

**Affiliations:** 1 Division of Geriatrics, Department of Medicine, McMaster University, Hamilton, Ontario, Canada

**Keywords:** Geriatrics, Medical education, Clinical clerkship, Activities of daily living, Patient outcome assessment, Clinical competence

## Abstract

**Background:**

The impact of geriatric medicine educational programs on patient level outcomes, as opposed to educational measures, is not well studied. We aimed to determine whether completion of a mandatory geriatrics rotation changed the clinical behaviors of clerks caring for older patients admitted to a medical clinical teaching unit.

**Methods:**

We reviewed the charts of 132 older (>70y) patients, admitted to one medical clinical teaching unit (CTU) during 2005, and cared for by a clinical clerk, for documented functional assessment, cognitive assessment, recognition of medications that cause confusion, and early removal of indwelling urinary catheters. Performance of these outcomes was compared between clerks who had completed a mandatory 2-week geriatrics rotation immediately before the medical CTU rotation (n = 62) and those who completed geriatrics immediately after (n = 74). Patient outcomes were also measured and compared between groups.

**Results:**

Compared to clerks without prior geriatric exposure, clerks with geriatrics exposure were almost 3 times as likely to assess function of their older patients within two days of assuming care (27% vs. 12%, OR: 2.73, 95% CI: 1.12 to 6.66). There were no significant differences in the other clinical behaviors. Patients cared for by geriatrics-exposed clerks were less likely to die or be institutionalized (10% vs. 31%, OR: 0.24, 95% CI: 0.09 to 0.63), and they had shorter lengths of stay by an average of -7.14 days (95% CI: -12.2 to -2.07). Adjustment for baseline differences in age and cognitive impairment did not alter the results.

**Conclusions:**

Clinical clerks who had completed a mandatory geriatrics rotation were more likely to document functional status upon assuming care of their older medical CTU patients, and there was also an association with better clinical outcomes. This highlights the value of including a geriatric medicine rotation as part of the core clerkship curriculum.

## Background

It is widely recognized that the number of frail elderly patients with complex health care needs is large and growing. Some medical schools have responded to this by introducing a mandatory clerkship rotation in geriatric medicine [1]. Educational programs in geriatric medicine have been shown to improve performance on knowledge tests, OSCE stations, faculty evaluations, attitudinal scales and self-assessment measures [2-8]. However, performance on paper does not necessarily translate into good patient care, and the impact of such educational programs on patient level outcomes is not known. Limited resources and skepticism about the added value of this type of experience have made it challenging to justify, implement and sustain mandatory geriatrics rotations. A deeper understanding of the ultimate clinical impact of educational programs in geriatric medicine would be very useful for educational planners.

The primary objective of this study was to answer the question: “Does completion of a mandatory geriatrics rotation change the clinical behaviors of clerks caring for older patients admitted to a medical CTU?”

## Methods

### Setting

Between 2002 and 2007, the clinical clerkship curriculum at McMaster University included a mandatory 2-week rotation in geriatrics. The core components included a half-day introductory workshop on comprehensive geriatrics assessment, and 2 weeks of clinical experience under the supervision of a geriatrician. Depending on the site, clerks spent time on geriatric assessment and rehabilitation units, inpatient consultation services, outpatient clinics, day hospitals or a combination of the above. There was variability in the acuity, and ambulatory content of clinical exposures, but all included experience working in an interdisciplinary model of care.

A typical team on the medical clinical teaching unit (CTU) rotation consists of an attending internist, a senior medical resident, 2–3 junior residents, and 2 medical students in the final year of the MD program - clinical clerks. Clinical clerks are the most junior members of the team, but are assigned individual patients and expected to take primary responsibility for their care. Their role includes daily medical assessment, progress notes, and reporting of their patients’ clinical status with proposed management plans, to senior members of the team. All clerks during the period of this study would have received approximately 14 hours of geriatrics-based content during the pre-clerkship curriculum.

The undergraduate medical education office assigned clerks, in no specific pattern, to complete the geriatrics rotation either immediately preceding or immediately following their 6-week rotation on a medical CTU. The undergraduate curriculum was otherwise not altered by the order of the assignment, and therefore the experimental conditions of a randomized controlled trial were approximated. This offered a unique opportunity to study the effect of a geriatrics rotation on the way clerks subsequently assessed and managed the older medical CTU patients that were assigned to them.

### Selection and description of participants

Participants met the following inclusion criteria: 1) age 70 or older, 2) admitted to the medical CTU at one acute care site during 2005, 3) length of stay of at least 4 days, and 4) majority of the admission spent on the medical CTU, geriatric assessment unit or alternate level of care ward. At the hospital where the study was conducted, patients were admitted first to the medical CTU, and if acute medical issues were resolved and the patient not immediately ready for discharge, they were transferred to the geriatric assessment unit or alternate level of care ward for rehabilitation or discharge planning. Of the resulting sample, 397 (52%) patients were randomly selected and their charts screened for evidence that a clerk had been responsible for their acute care management for at least 2 consecutive days. A total of 136 clerk-patient encounters were obtained for evaluation. Approval for the study was obtained from the St. Joseph’s Healthcare Hamilton Research Ethics Board.

### Demographic and descriptive data collected

Data was abstracted from the medical records by one author (JS), who was blinded to the history of clerk geriatrics exposure. For each patient, age, gender, reasons for admission, chronic co-morbid conditions documented at the time of admission, complications arising during the hospitalization, and living environment (home, assisted living/retirement home, long term care) prior to admission and post-discharge, were recorded. Charlson Comorbidity Index was calculated according to the method described by Charlson [9]. For each clinical clerk, number of days from the start of clinical clerkship was calculated as a measure of experience. For ethical reasons, clerk characteristics such as gender, career choice, and academic ability could not be determined. After data collection was completed, dates of service for each clerk-patient encounter were cross-referenced to the 2005 clerkship schedule to determine whether a geriatrics rotation had been previously completed or not. There was no temporal overlap between the 6-week medical CTU rotations; therefore, dates of service confidently identified the rotation history for each clerk-patient encounter.

### Outcome measures

Only progress notes and orders written by the clinical clerk, during the time frame that the clerk was on service, were examined. The primary outcome of interest was assessment of functional status within 2 days of assuming care. This was defined as documentation of at least one activity of daily living or a summary statement of current or pre-admission functional status. Assessment of cognition within 2 days of assuming care was defined as any documentation related to cognitive status, either descriptive (e.g. “oriented X 3”) or quantitative (e.g. MMSE score). Recognition that sedative, opioid, and anticholinergic medications may cause confusion was considered to have occurred when a progress note mentioned the possible cognitive risks of these agents. The duration of indwelling catheter was measured from the first day that the patient had a catheter while under the clerk’s care, to the date an order was written for removal.

These outcomes were chosen because they have high relevance for the optimal care of older patients on acute medical units, and they reflect the Core Competencies in the Care of Older Persons for Canadian Medical Students as defined by the Medical Education Committee of the Canadian Geriatrics Society [10]. They are also consistent with Minimum Geriatrics Competencies for Medical Students defined by the 2007 Association of American Medical Colleges/John Hartford Foundation Consensus Conference [11], and quality of care indices as defined by the Assessing Care of Vulnerable Elders (ACOVE) project [12]. Referrals to geriatric medicine or occupational therapy during the period of clerk involvement and total patient length of stay, mortality and discharges to long-term care (LTC) were also recorded. The combined endpoint of death or new discharge to LTC was considered as a composite adverse clinical outcome.

### Analysis

The patient populations were defined using descriptive statistics. Continuous and categorical variables were compared using the Student t-test and Fisher exact test, respectively. The clinical clerkship experience variable was not normally distributed thus a Wilcoxon Rank-Sum test was performed to determine differences between groups. Primary and secondary outcome measures were compared between the patients cared for by students with prior geriatrics exposure (GM exposure) versus those students without prior geriatrics exposure (No GM exposure). Odds ratios with adjusted 95% confidence intervals were calculated for the binary outcome measures, and adjusted for possible confounders (either age or cognitive impairment) using multivariable logistic regression analysis. For length of stay, parameter estimates were calculated using multivariable linear regression analysis, and adjusted for potential confounders (either age or cognitive impairment). All statistical analyses were performed using the SAS/STAT (version 9.2; SAS institute Inc., Cary, North Carolina, USA) software package running on Window XP Professional. Two-tailed P-values of less than 0.05 were considered to indicate statistical significance.

## Results

Demographics of the patients, and clinical experience of the clerks looking after them are outlined in Table 1. Both patient groups were well matched in terms of gender and Charlson Comorbidity Index. Both groups included patients with a broad range of conditions that are commonly seen on general medical wards [13]. Patients cared for by the clerks without geriatrics exposure were slightly older, less likely to come from long term care institutions, more likely to have a prior diagnosis of cognitive impairment, and more likely to have an admitting diagnosis of delirium or confusion. They were also more likely to have acute coronary syndrome, and less likely to have chronic lung disease listed as a reason for admission. Length of clinical clerkship experience prior to the observation period did not differ significantly between groups.

**Table 1:** Baseline characteristics according to clerkship group

***Characteristic***	***GM exposure***	***No GM exposure***	***P-value***
	***(n = 62)***	***(n = 74)***	
***Patient characteristics***			
Age, mean (SD)	79.92 (6.02)	82.76 (7.07)	0.01
Female, (%)	61.29	52.70	0.39
Residence prior to admission, (%)			
Home	77.42	81.08	0.67
Assisted living/retirement home	6.45	13.51	0.26
Long term care	16.13	5.41	0.05
Charlson comorbidity index, mean (SD)	2.69 (1.84)	2.40 (1.62)	0.33
Admitting diagnosis, (%)			
Pneumonia/influenza	20.97	22.97	0.84
Heart failure	14.52	14.86	1.00
Chronic lung disease	14.52	2.70	0.02
Urinary tract infection	9.68	6.76	0.55
Acute coronary syndrome	0.00	9.46	0.02
Atrial fibrillation	9.68	6.76	0.55
Falls	8.06	12.16	0.57
Delirium/confusion	1.61	12.16	0.02
Weakness	6.45	9.46	0.75
Failure to cope	6.45	2.70	0.41
Comorbidities, (%)			
Diabetes mellitus	37.10	22.97	0.09
Coronary artery disease	30.65	32.43	0.86
Chronic lung disease	27.42	14.86	0.09
Arrhythmia	22.58	25.68	0.69
Cognitive impairment	8.06	25.68	0.01
Heart failure	22.58	21.62	1.00
Cerebrovascular disease	20.97	21.62	1.00
Psychiatric disorder	14.52	9.46	0.43
Chronic renal failure	14.52	8.11	0.28
***Clerk characteristics***			
Experience in days, median (IQR)	181 (30–264)	114 (28–201)	0.37

Tables 2 and 3 summarize the results. Compared to clerks without prior geriatrics exposure, clerks who had recently completed the geriatrics rotation were almost 3 times as likely to assess function of their older patients within 2 days of assuming care (Table 2). This relationship persisted after adjusting for patient age or comorbid cognitive impairment. No significant differences between groups were found in assessment of cognition, recognition of harmful medications, use of catheters, or referral rates to geriatric medicine or occupational therapy.

**Table 2:** Differences in clinical behaviors of clerks according to history of geriatric medicine (GM) exposure

**Outcome**	**GM exposure**	**No GM exposure**	**GM exposure versus No GM exposure**		
	**(n = 62)**	**(n = 74)**			
	**n (%)**	**n (%)**	**Odds ratio (95% CI)**		
			**Unadjusted**	**Adjusted for age**	**Adjusted for cognitive impairment**
Functional assessment within 2 days	17 (27.42)	9 (12.16)	2.73 (1.12-6.66)	2.71 (1.09-6.74)	3.02 (1.18-7.73)
Cognitive assessment within 2 days	29 (46.77)	36 (48.65)	0.93 (0.47-1.82)	1.08 (0.53-2.17)	1.10 (0.54-2.22)
Identified medications that may cause confusion^1^	5 (14.29)	5 (18.51)	0.73 (0.19-2.85)	Not applicable^2^	Not applicable^2^
Catheter used for <3 days^3^	13 (48.15)	12 (37.50)	1.55 (0.55-4.38)	1.12 (0.36-3.45)	1.26 (0.42-3.77)
Referred to geriatric medicine	8 (12.90)	9 (12.16)	1.07 (0.39-2.96)	1.06 (0.37-2.99)	1.07 (0.38-3.06)
Referred to occupational therapy	25 (40.32)	24 (32.43)	1.41 (0.70-2.84)	1.49 (0.73-3.08)	1.68 (0.80-3.51)

^1^As a percent of patients who were prescribed sedative, opioid, or anticholinergic medications.

^2^Numbers too small for analysis.

^3^As a percent of patients with urinary catheters inserted.

**Table 3:** Differences in patient outcomes according to history of geriatric medicine exposure

**Outcome**	**GM exposure**	**No GM exposure**	**GM exposure versus No GM exposure**		
	**(n = 62)**	**(n = 74)**			
	**n (%)**	**n (%)**	**Odds ratio (95% CI)**^**a**^		
			**Unadjusted**	**Adjusted for age**	**Adjusted for cognitive impairment**
Death or new discharge to LTC	6 (9.68)	23 (31.08)	0.24 (0.09,0.63)	0.30 (0.11,0.85)	0.27 (0.10,0.73)
	**Mean (SD)**	**Mean (SD)**	**Parameter estimate (95%CI)**^**b**^		
			**Unadjusted**	**Adjusted for age**	**Adjusted for cognitive impairment**
Length of stay, days	15.95 (11.68)	23.09 (17.14)	-7.14 (-12.2, -2.07)	-6.38 (-11.55, -1.21)	-6.24 (-11.4, -1.05)

^a^Determined by logistic regression analysis.

^b^Determined by linear regression analysis.

Patients cared for by the geriatrics-exposed clerks were much less likely to experience the composite adverse event of death or institutionalization and they had shorter lengths of stay by one week (Table 3). These differences also persisted after adjusting for age and cognitive impairment.

## Discussion

This study found that clinical clerks who had completed a mandatory geriatrics rotation were more likely to document the functional status of older patients during their medical CTU rotation. Functional decline commonly accompanies hospitalization in the elderly, increases directly with age [14], and is an important determinant of discharge planning, healthcare costs, and clinical outcomes [15]. The ability to recognize, prevent and manage functional loss has therefore become an indispensable competency in acute care settings. In this study, length of stay, survival and avoidance of institutionalization, were more favorable in the patient group cared for by geriatrics-exposed clerks. This relationship persisted, even when corrected for age or cognitive impairment. There was no compensatory increase in referrals to other services that could address function (e.g. occupational therapy, geriatric medicine), indicating that the patients assigned to clerks without geriatrics exposure truly received less attention to functional issues.

Based on these results, the authors hypothesize that awareness and management of functional impairments may have contributed to improved patient outcomes and more efficient discharges. Functional assessment is probably not emphasized in other areas of the medical curriculum because it does not contribute to traditional diagnostic algorithms. A core principle of geriatric medicine is that functional status has critical implications for how one assesses and manages frail older patients, and it may be that this learning point can only be fully appreciated when observed in real clinical contexts.

Clinical clerks require cosignature for all orders and are perceived to have limited influence on patient management. However, they may still impact patient care by recognizing easily overlooked problems and suggesting low risk, high benefit interventions. For example, in the frail elderly, rapid functional decline is often a more reliable indicator of occult illness than reported symptoms, physical exam or lab abnormalities. A clerk with geriatrics training would have learned to assess and investigate further when a patient manifests unexpected functional decline. The geriatrics rotation may have also empowered clerks to advocate for optimizing the care of patients that otherwise might be viewed as lacking potential for recovery.

Completion of the geriatrics rotation had no significant impact on the other behavioral outcomes, and there are several possible reasons for this. In a two-week exposure, certain clinical scenarios (e.g. indwelling catheters, harmful psychoactive medications) may occur too infrequently to expect a significant impact on learning. For other competencies that have adequate coverage in other parts of the curriculum (e.g. diagnostic approach to cognitive impairment), a clinical experience in geriatrics may not have much added impact.

Most studies in medical education have examined intermediate educational outcomes (e.g. acquisition of knowledge, learner satisfaction, faculty evaluation) rather than patient-level outcomes, and this is considered a gap in medical education research [16-18]. A content analysis of leading medical education journals found that patient outcomes were examined in only 0.7% of articles [19]. Good performance on educational measures is assumed to translate into quality patient care; however, this is not necessarily the case. Ageism exists in clinical teaching environments, and can lead to therapeutic nihilism or substandard care despite adequate knowledge. This would not be captured in studies that look solely at measures such as attitudinal scales or tests of knowledge. This study shows that it is feasible and worthwhile to conduct educational research examining impact on patient level outcomes.

There are several limitations to this study. First, there were some baseline differences between patient groups, and these or other unmeasured variables may have confounded the results. Clerks with geriatrics exposure were more likely to be assigned patients that were younger, and cognitively intact. This patient group may have been easier to interview for functional history and more likely to have positive clinical outcomes. Our analysis controlled for age and cognitive impairment, but other variables such as frailty [20] and falls risk [21] have also been shown to affect patient outcomes, and these were not measured. Second, clinical clerks will typically confer with other clinical team members before documenting patient decisions. Therefore, documented care may not be directly attributed to a single clerk but to the clinical team. The degree of outside influence on clerk behaviors in this study cannot be determined. However, functional assessment is not likely to be questioned or modified by supervisors, and we think the potential for outside influence on this particular outcome is low. Third, the study occurred at a single teaching site, with a specific patient population, therefore may not be generalizable to other clinical teaching settings. Finally, the study does not tell us whether improved assessment of function was sustained beyond six weeks, or in subsequent clinical experiences.

A total of 820 students participated in the mandatory geriatrics clerkship. A small and static number of geriatric medicine specialists at our institution, coupled with increased medical school enrolment over the past decade eventually made this an unsustainable model. In 2007, the mandatory geriatrics clerkship was changed to an elective rotation. Only 20% of the class will participate in a geriatrics rotation in 2014. Some argue that geriatrics content is already adequately integrated into the internal medicine clerkship because most inpatients are old. However, like Diachun and colleagues [8], we demonstrated that there are unique competencies that are not taught by non-geriatrics specialists, even if they provide care for a predominantly aged population.

## Conclusions

Despite current demographic trends, medical education remains focused on younger populations and conditions that are more acute, rare, high-tech and curative. Those who work in geriatrics routinely witness ageism within our health care system and clinical teaching environments [22], making it a challenging area to achieve uptake of educational efforts. Teaching about specific diseases often takes precedence over fostering important, practical behaviors like assessing, preventing and responding to functional loss. This study shows that clinical exposure to geriatric medicine is strongly associated with improved attention to functional assessment and improved clinical outcomes at the level of patient care.

Medical education must be publically accountable and appreciate how it can make a positive impact on health care outcomes. Quality of care for geriatric conditions is demonstrated to be worse than for other medical conditions [23], therefore effective educational programs in geriatric medicine are needed. The suboptimal care and poor outcomes of older patients in acute care settings is often viewed predominantly as a systems or social problem, deflecting responsibility from the physicians. We hope that this study will convince medical education planners that a clinical clerkship experience specifically dedicated to geriatric medicine, and relatively brief exposure to practicing geriatricians is a valuable component of the undergraduate curriculum.

## Abbreviations

ALC: Alternate level of care; CI: Confidence interval; CTU: Clinical teaching unit; GAU: Geriatric assessment unit; GM: Geriatric medicine; IQR: Interquartile range; OR: Odds ratio; SD: Standard deviation.

## Competing interests

The authors have no conflicts of interest to declare.

## Authors’ contributions

JS conceived of the study, collected data, and drafted the manuscript. AP helped with study design, data interpretation and manuscript editing. GI performed statistical analysis, helped with study design and manuscript editing. HM & SM helped with study design, and manuscript editing. All authors read and approved the final manuscript.

## Pre-publication history

The pre-publication history for this paper can be accessed here:

http://www.biomedcentral.com/1472-6920/13/168/prepub
